# Research on the Modality Transfer Method of Brain Imaging Based on Generative Adversarial Network

**DOI:** 10.3389/fnins.2021.655019

**Published:** 2021-03-15

**Authors:** Dapeng Cheng, Nuan Qiu, Feng Zhao, Yanyan Mao, Chengnuo Li

**Affiliations:** ^1^School of Computer Science and Technology, Shandong Technology and Business University, Yantai, China; ^2^Shandong Co-Innovation Center of Future Intelligent Computing, Yantai, China

**Keywords:** brain imaging modality transfer, EEG, fMRI, generative adversarial network, non-adversarial loss

## Abstract

Brain imaging technology is an important means to study brain diseases. The commonly used brain imaging technologies are fMRI and EEG. Clinical practice has shown that although fMRI is superior to EEG in observing the anatomical details of some diseases that are difficult to diagnose, its costs are prohibitive. In particular, more and more patients who use metal implants cannot use this technology. In contrast, EEG technology is easier to implement. Therefore, to break through the limitations of fMRI technology, we propose a brain imaging modality transfer framework, namely BMT-GAN, based on a generative adversarial network. The framework introduces a new non-adversarial loss to reduce the perception and style difference between input and output images. It also realizes the conversion from EEG modality data to fMRI modality data and provides comprehensive reference information of EEG and fMRI for radiologists. Finally, a qualitative and quantitative comparison with the existing GAN-based brain imaging modality transfer approaches demonstrates the superiority of our framework.

## 1. Introduction

Brain imaging modality transfer has become popular in the field of medical imaging, providing a variety of reference information for early diagnosis, identification, treatment, and follow-up of the diseases (Yi et al., 2019). For example, the translation of computed tomography (CT) modality data to magnetic resonance (MR) modality data (Jin et al., 2019) solves the problem of lack of MR modality data or synthesizes positron emission tomography (PET) modality data from CT modality data (Choi and Lee, 2018) for cancer staging, detection, and treatment.

The modality transfer of brain imaging is to map one modality to another (Armanious et al., 2020). The information provided by an image obtained from a certain imaging method is often limited, and can only reflect the information of one modality, which generally cannot help the doctor to make an accurate diagnosis. The modality transfer technology is conducive to converting between different modality images to obtain multi-modality information. Combined with multi-modality images, it can provide a variety of information about diseased tissues or organs, and provide a strong theoretical basis for clinical medicine to make an accurate diagnosis. As for the modality transfer of brain imaging, the current research at home and abroad is mainly based on the image-to-image translation. Existing image-to-image translation methods are mainly divided into two categories: sparse representation-based method and learning-based method.

The method based on sparse representation (SR) is the process of using image block prediction. An image block is extracted from an atlas with the same modalities as the source image to sparsely represent the source image, and then the obtained sparse coefficient is used to estimate the target image block of another modality image. Ye et al. (2013) used T1-weighted MR images to synthesize T2-weighted MR images. Cordier et al. (2016) used brain labeled images to predict FLAIR images. However, the sparse representation-based approach requires sparse coding optimization of image blocks at all locations, which will lead to a decrease in the efficiency of the prediction process, and sometimes there will be noise in the synthesis results.

The convolutional neural network (CNN) in the “two networks” shows good performance in the fields of computer vision and medical image analysis. Li et al. (2014) used CNN to predict PET images from MR. Han (2017) used deep CNN to synthesize CT with MR. But, the training of a robust CNN model requires a large data set and a long training time. However, there is an unavoidable problem in the CNN-based image modality migration task. Although the entire network training process is automatic, it still needs to design loss function, namely, design loss network parameters.

Since the GAN concept was proposed, it has been showing strong practicability in image synthesis. The work of Denton et al. (2015) and Salimans et al. (2016) has proved that GAN has been showing strong practicability in the image. GAN can solve the problems encountered in CNN well. According to the work of Zhao et al. (2016), it is proved that when using CNN, a specific loss function is needed to deal with the problem, and GAN can achieve good results. At the same time, a new GAN network structure—Conditional GANs was proposed, referring to the design pattern of the CNN network and adding Settings for specific applications when GAN was used. A lot of work confirmed that such a GAN network has a good effect in processing images, videos, and 3D data. Isola et al. (2017) have proposed an image translation structure Pix2Pix based on conditional GAN, which can achieve good results under supervised learning.

However, a network based on supervised learning requires paired data training, and patients should be tested for both EEG and fMRI at short intervals. Besides, further, post-processing is required, such as image registration (Yang et al., 2018). Obtaining these data is a great challenge. Whatsmore, with the development of computer technology and digital imaging technology, brain imaging methods are gradually mature and diversified, yet many of these technologies still have limitations. For example, functional magnetic resonance imaging types of equipment are expensive, and patients who use pacemakers cannot use the fMRI. Although fMRI can accurately locate the active area when “observing the active brain” and its spatial resolution can reach the millimeter level (Tang et al., 2019), its temporal resolution is far lower than EEG (Menon et al., 1998). In contrast, EEG is easier to implement. Thus, if we can realize the conversion from EEG to fMRI, we can obtain both advantages of EEG and fMRI by implementing only one of them. However, to our best knowledge, few studies have explored the conversion of EEG to fMRI modality data.

In this work, we propose a brain imaging modality transfer framework, which uses 2D sensor cap images of EEG and T1-weighted axial images from fMRI to infer the target fMRI modality data, providing comprehensive reference information for medical diagnosis. Specifically, we focus on generating fMRI modality data from EEG modality data, which requires learning the mapping relationship between these two different modality data. However, it is well-known that converting between different modality data has always been a challenge. Fortunately, the emergence of the generative adversarial network (GAN) (Goodfellow et al., 2014) improves this aspect. GAN has achieved advanced performance in learning the mapping between different modalities. Meanwhile, with the development of deep learning, GAN has become one of the hot research directions in the field of medical imaging.

Therefore, we leverage GAN to achieve a brain imaging modality transfer framework named BMT-GAN. This framework introduces the combination of cycle-consistency loss and adversarial loss used in the CycleGAN framework proposed by Wolterink et al. (2017). which avoids the inability of traditional GAN to achieve pairing problems between input and output. Besides, the framework introduces non-adversarial loss, which reduces the perception and style differences between input and output modality images, and enhances the global consistency between input and output images. A qualitative and quantitative comparison with the existing GAN-based medical image modality transfer methods proves that BMT-GAN can effectively capture the overall tissue contrast and local anatomical details of fMRI images.

The contributions of this article are summarized as follows:

(1) We proposed a new framework named BMT-GAN to estimate two-dimensional fMRI images using two-dimensional EEG images;

(2) BMT-GAN framework combines the cycle-consistent loss, adversarial loss, and non-adversarial loss to achieve excellent brain imaging modality transfer performance;

(3) The proposed approach can be easily extended to other medical data translation tasks to benefit the medical imaging field.

## 2. Methods

**Figure 1:**
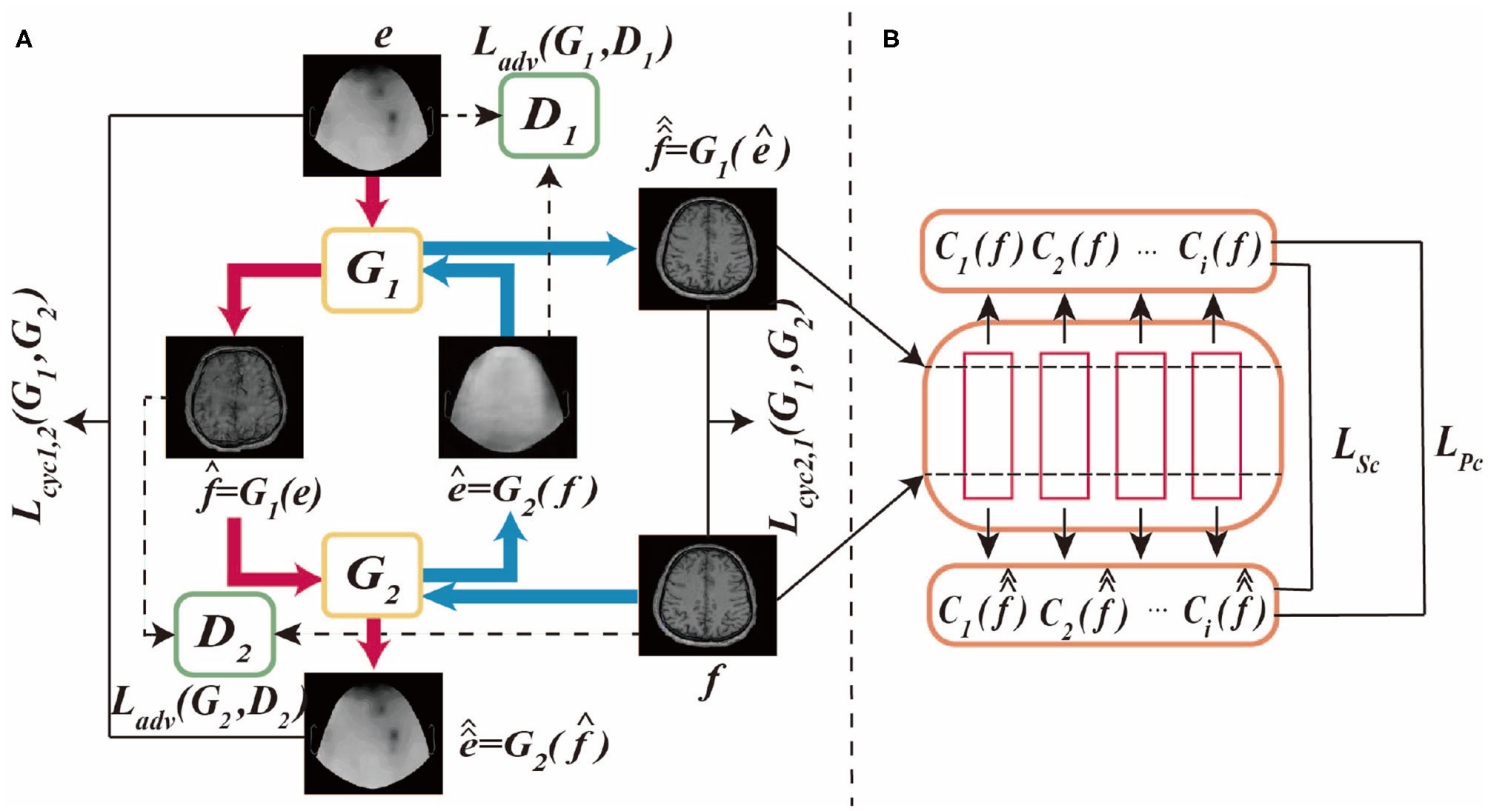
This is BMT-GAN framework, **(A)** is CycleGAN framework, **(B)** is non-adversarial structure. The CycleGAN framework allows bidirectional translation between fMRI images and EEG images. e and f are unpaired images randomly sampled from their respective domains. The non-adversarial structure includes feature-based perceptual loss and style loss functions *L*_*Pc*_ and *L*_*Sc*_.

The proposed BMT-GAN framework utilizes the dual thought (Farnia and Tse, 2018) of Welander et al. (2018), which not only adopts the cycle-consistency loss and adversarial loss of CycleGAN model but also introduces a new non-adversarial loss combination. Whatsmore, Figures 1A,B shows the BMT-GAN framework structure of two parts: CycleGAN and non-adversarial structure.

### 2.1. CycleGAN

CycleGAN is an unsupervised framework that allows image-to-image translation without matching training data samples in two domains. The network framework structure and data flow diagram are shown in Figure 1A.

As shown in Figure 1A, the differences between CycleGAN framework and traditional GAN are: (1) CycleGAN consists of two generators (*G*_1_, *G*_2_) and two discriminators (*D*_1_, *D*_2_); (2) CycleGAN instead of learning a single mapping, it learns two mapping functions between two domains: *G*_1_:*I*_*EEG*_ → *I*_*fMRI*_ and *G*_2_:*I*_*fMRI*_ → *I*_*EEG*_, ensuring that the input and output images can be matched after translation. Where, *I*_*EEG*_ is the sample space of the given EEG image training data, *e* ∈ *I*_*EEG*_, *I*_*fMRI*_ is the sample space of the given fMRI image training data, *f* ∈ *I*_*fMRI*_.

The framework of CycleGAN consists of two major branches *Cyc*_1,2_ and *Cyc*_2,1_. In the generation network of the *Cyc*_1,2_ branch, the generator *G*_1_ takes the EEG images in the source domain *I*_*EEG*_ as input, and outputs the synthetic translation $\hat{f}=G_{1}\left(e\right)$. To prevent a group of different images from being mapped to a single image in the target domain, the model requires the two generators to keep the loop consistent with each other. Therefore, using $\hat{f}$ as the input of *G*_2_, the output reconstruction $\hat{\hat{e}}=G_{2}\left(\hat{f}\right)$ is obtained. In the *Cyc*_2,1_ branch, the discriminator uses the fMRI images of the target domain *I*_*fMRI*_ as the input of *G*_2_, and the synthesized translation ê = *G*_2_(*f*). In the same way, using ê as the input of *G*_1_ to get the output reconstruction $\hat{\hat{f}}=G_{1}\left(\hat{e}\right)$. Therefore, the cycle-consistent loss of the two branches can be formulated as:

(1) $$\begin{array}{l} \begin{array}{c} L_{Cyc_{1,2}}\left(G_{1},G_{2}\right)=E_{e\in I_{EEG}}\left[||G_{2}\left(G_{1}\left(e\right)\right)-e|{|}_{1}\right]\\ L_{Cyc_{2,1}}\left(G_{2},G_{1}\right)=E_{f\in I_{fMRI}}\left[||G_{1}\left(G_{2}\left(f\right)\right)-f|{|}_{1}\right] \end{array} \end{array}$$

Also, each generator uses a corresponding discriminator for adversarial training. In the discriminator of the *Cyc*_1,2_ branch, the fMRI image in the target domain *I*_*fMRI*_ and the synthetic translation $\hat{f}$ are used as the *D*_1_ input of the discriminator. In the *Cyc*_2,1_ branch, the EEG image in the source domain *I*_*EEG*_ and the synthetic translation ê from the source domain are used as the input of *D*_2_. Among them, *D*_1_ and *D*_2_ act as binary classifiers to distinguish the converted image from the target domain image as accurately as possible. Besides, the generator synthesizes high-quality images through iterative training to obfuscate the discriminator. Therefore, the model's adversarial loss function can be formulated as:

(2) $$\begin{array}{l} L_{Adv_{1,2}}\left(G_{1},D_{1}\right)=E_{f\in I_{fMRI}}\left[logD_{1}\left(f\right)\right]\\ \;+E_{e\in I_{EEG}}\left[log\left(1-D_{1}\left(G_{1}\left(e\right)\right)\right)\right]\\ L_{Adv_{2,1}}\left(G_{2},D_{2}\right)=E_{e\in I_{EEG}}\left[logD_{2}\left(e\right)\right]\\ \;+E_{f\in I_{fMRI}}\left[log\left(1-D_{2}\left(G_{2}\left(f\right)\right)\right)\right] \end{array}$$

### 2.2. Non-adversarial Structure

In order to ensure that the input and output images can be paired after image translation, the loss functions in Equations (1) and (2) are introduced. However, during the training process, due to the large differences in the anatomical details of the 2D EEG image and the 2D fMRI image, the pixel-level loss becomes ineffective and the output translation image lacks clarity and fine structure (Johnson et al., 2016; Wang C. et al., 2018). For example, Jin et al. (2019) used a combination of adversarial loss and the traditional L1 distance loss. This loss combination has achieved good results in the translation task of paired images. Armanious et al. (2020) used a combination of perceptual loss and style loss in addition to cycle-consistent and adversarial loss in the MedGAN framework, and also achieved success.

However, for unpaired image translation tasks, it is not feasible to use the above two-loss combinations due to the huge difference between the source domain image and the target domain image. Therefore, we introduce a feature-based loss function between input reference and output reconstruction as an additional constraint to improve output quality. The process is shown in Figure 1B. The first proposed loss function is the cycle-perceptual loss, *L*_*Pc*_. This is achieved by extracting intermediate feature maps using a pre-trained feature extractor network, for both the input and the cycle-reconstructed images. This process is formulated as a non-adversarial perceptual loss function *L*_*Pc*_:

(3) $$\begin{array}{l} L_{Pc}=\sum_{i=0}^{N}\omega_{pc,i}(||C_{i}(e)-C_{i}(\hat{\hat{e}})|{|}_{1}+||C_{i}(f)-C_{i}(\hat{\hat{f}})|{|}_{1}) \end{array}$$

Where *C*_*i*_ is the feature map extracted by the *ith* layer of the feature extractor network. N is the total number of layers, and ω_*pc,i*_ is the weight of each layer.

Secondly, a style-based loss function between input reference and output reconstruction is proposed to compensate for the difference in style representation between the reconstructed image and its corresponding target image. By calculating the relevance of the feature representation in the spatial range, the style distribution can be captured. To calculate the style loss,we must first calculate *G*_*r*_*i*__(*e*):

(4) $$\begin{array}{l} G_{r_{i}}{\left(e\right)}_{m,n}=\frac{1}{h_{i},w_{i},d_{i}}\overset{h_{i}}{\underset{h=1}{\sum }}\overset{w_{i}}{\underset{w=1}{\sum }}C_{i}{\left(e\right)}_{h,w,m}C_{i}{\left(e\right)}_{h,w,m} \end{array}$$

Where *G*_*r*_*i*__(*e*) is the Gram matrix of each convolution block, the shape is *d*_*i*_ *d*_*i*_, which is used to express the feature association. Its elements are obtained by inner product calculation on the height and width of the feature graph. *h*_*i*_, *w*_*i*_, *d*_*i*_ the height, width, and depth of the feature space. The non-adversarial style loss function is formulated as *L*_*Sc*_:

(5) $$\begin{array}{l} L_{Sc}=\sum_{i=0}^{N}\omega_{sc,i}\frac{1}{4d_{i}^{2}}(||G_{r_{i}}(e)-G_{r_{i}}(\hat{\hat{e}})|{|}_{F}^{2}+||G_{r_{i}}(f)-G_{r_{i}}(\hat{\hat{f}})|{|}_{F}^{2}) \end{array}$$

ω_*sc,i*_ is the ith weight of the given Gram matrix.

Therefore, the ultimate minimum and maximum optimization task of the BMT-GAN framework is formulated as follows:

(6) $$\begin{array}{l} \begin{array}{c} \underset{G_{1},G_{2}}{\min}\underset{D_{1},D_{2}}{\max}L=L_{Cyc_{1,2}}\left(G_{1},G_{2}\right)+L_{Cyc_{2,1}}\left(G_{2},G_{1}\right)+L_{Adv_{1,2}}\left(G_{1},D_{1}\right)\\ +L_{Adv_{2,1}}\left(G_{2},D_{2}\right)+\omega_{pc}L_{Pc}+\omega_{sc}L_{Sc} \end{array} \end{array}$$

### 2.3. Generator and Discriminator

**Figure 2:**
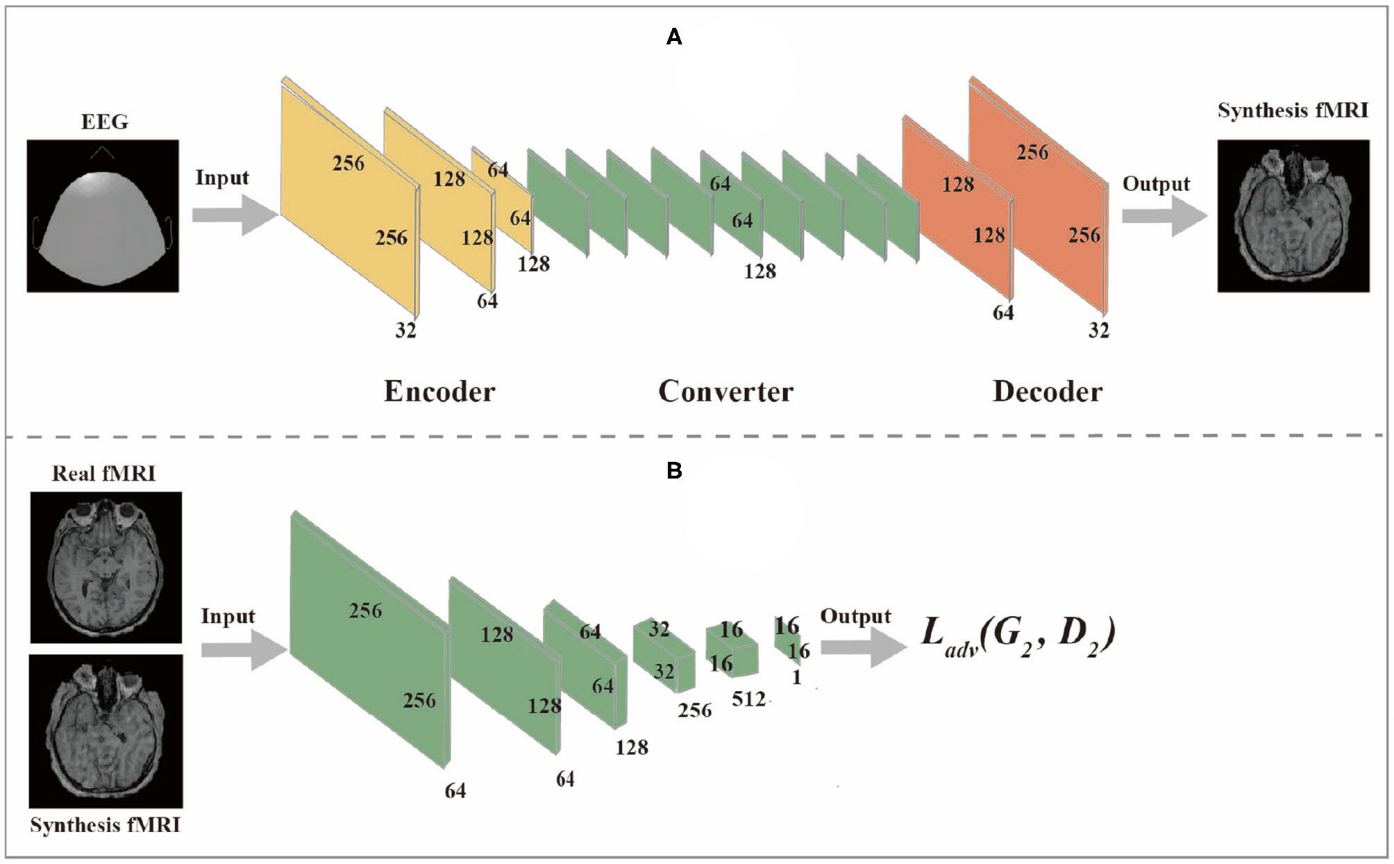
**(A)** is Generator network, **(B)** is Discriminator network.

The generator network is composed of three components: encoder, converter, and decoder, while the discriminator network is composed of multiple convolutional layers, the flow diagram as shown in Figures 2A,B.

#### 2.3.1. Generator Network

(1) The encoder is composed of three convolutional layers. The initial size of the input image is 256 × 256 × 1. The first step is to extract features from the image through the convolutional layer. The number of features extracted from the convolutional layer can also be regarded as the number of different filters used to extract different features. The convolutional layer gradually extracts more advanced features in turn. After passing through the encoder, the input image changes from (256,256,1) to (64,64,128).

(2) The converter is composed of nine residual blocks. Different features of the image are combined by different channels of the output image of the encoder. According to these features, the feature vector of the image is converted from the source domain to the target domain. However, since the images in the EEG domain and fMRI domain do not have similar features, the feature vectors of images in the EEG domain should be converted into feature vectors in the fMRI domain through the sharing function of the generator during the conversion process. The residual block consists of two convolutional layers and the input residuals are added to the output to ensure that the input properties of the previous layer can also be applied to the later layers so that their output will not be different from the original input. Otherwise, the features of the original image will not be retained in the output.

(3) The role of a decoder is to reconstruct low-level features from feature vectors, which can be accomplished by using a deconvolution layer. Finally, the low-level functions are converted to images in the target domain.

#### 2.3.2. Discriminator Network

The discriminator takes an image as input and predict whether the image is the original image or the output of the generator. The discriminator is composed of multiple convolutional layers. After extracting features from the image, the discriminator can determine whether these features belong to a specific category. The last layer of the discriminator network is the convolutional layer used to generate one-dimensional output.

## 3. Experiment and Results

We compared the BMT-GAN framework with several state-of-the-art brain imaging modality translation approaches. In this section, we will describe the dataset, model performance evaluation, evaluation metrics, qualitative, and quantitative results to demonstrate the effectiveness of the brain imaging modality migration approach.

### 3.1. Datasets

**Figure 3:**
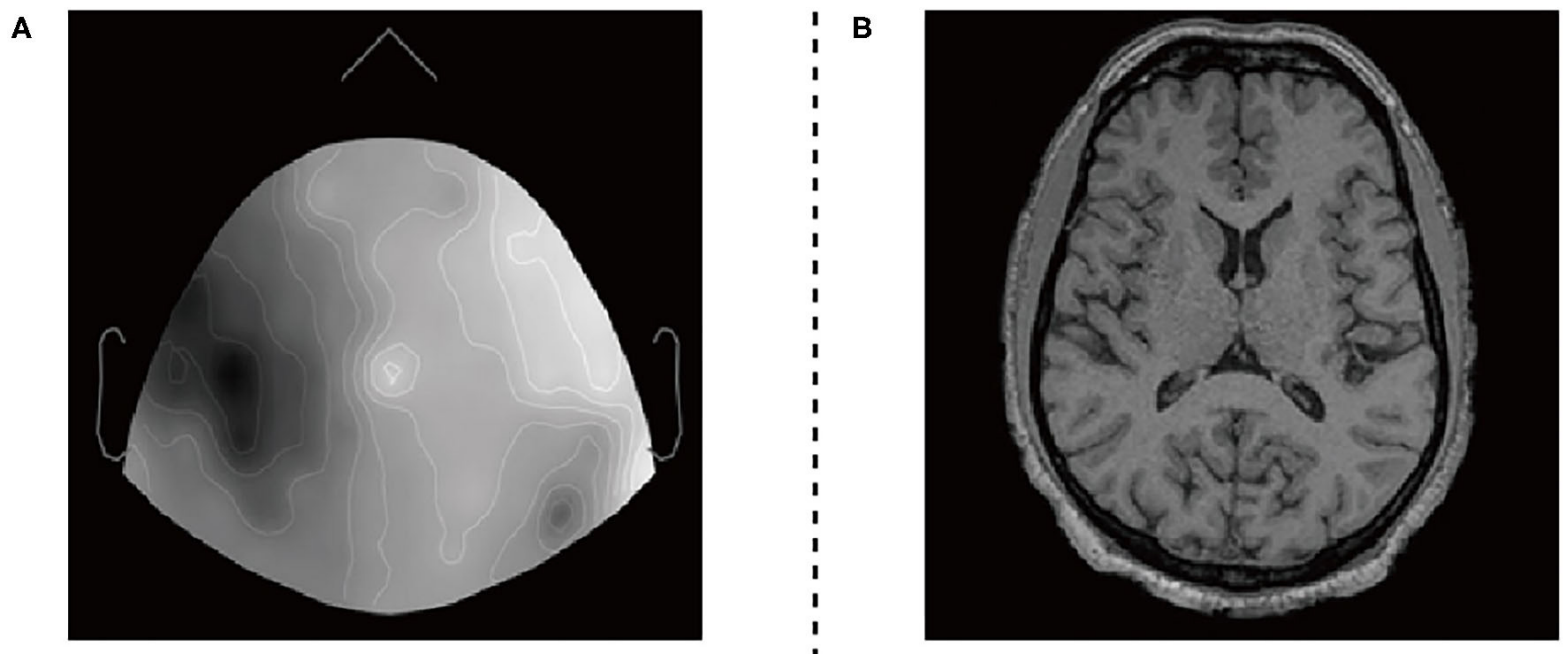
EEG datasets and fMRI datasets. **(A)** 2D sensor cap image of EEG. **(B)** T1 axial slice image of fMRI.

In our work, we applied the two-dimensional sensor cap image of EEG and the two-dimensional axial slice of the high-resolution T1-weighted image of fMRI. The utilized datasets are illustrated in Figure 3.

The EEG and fMRI imaging data from 17 adult volunteers. They were acquired in a Siemens Avanto 1.5T clinical scanner using a self-shielded gradient set with maximum gradient amplitude of 40*m*^*T*^*m*^−1^, derived from Deligianni et al. (2014). The fMRI imaging data acquisition was based on a *T*2* -weighted gradient-echo EPI sequence with 300 volumes, and the effective voxel size is 3.3 × 3.3 × 4.0*mm*^3^. Scalp EEG was recorded during the MRI scan using a 64-channel MR-compatible electrode cap (BrainCap MR, Gilching, Germany) at a native frequency of 1,000 Hz.

The two types of imaging data were visualized by using Brainstorm visualization software. Each volunteer involved 35 or more EEG and fMRI two-dimensional axial slices. They have pixels of the same size as 256 × 256 × 1. We divide the datasets into two training sets and two test sets. The training set includes EEG images and fMRI images of 12 subjects, and the test set includes EEG images and fMRI images of the remaining subjects. Secondly, we used the Intel(R) Core(TM) i7-9700 CPU processor for 2*e*^5^ iterations. The entire training process takes about 40 h.

**Figure 4:**
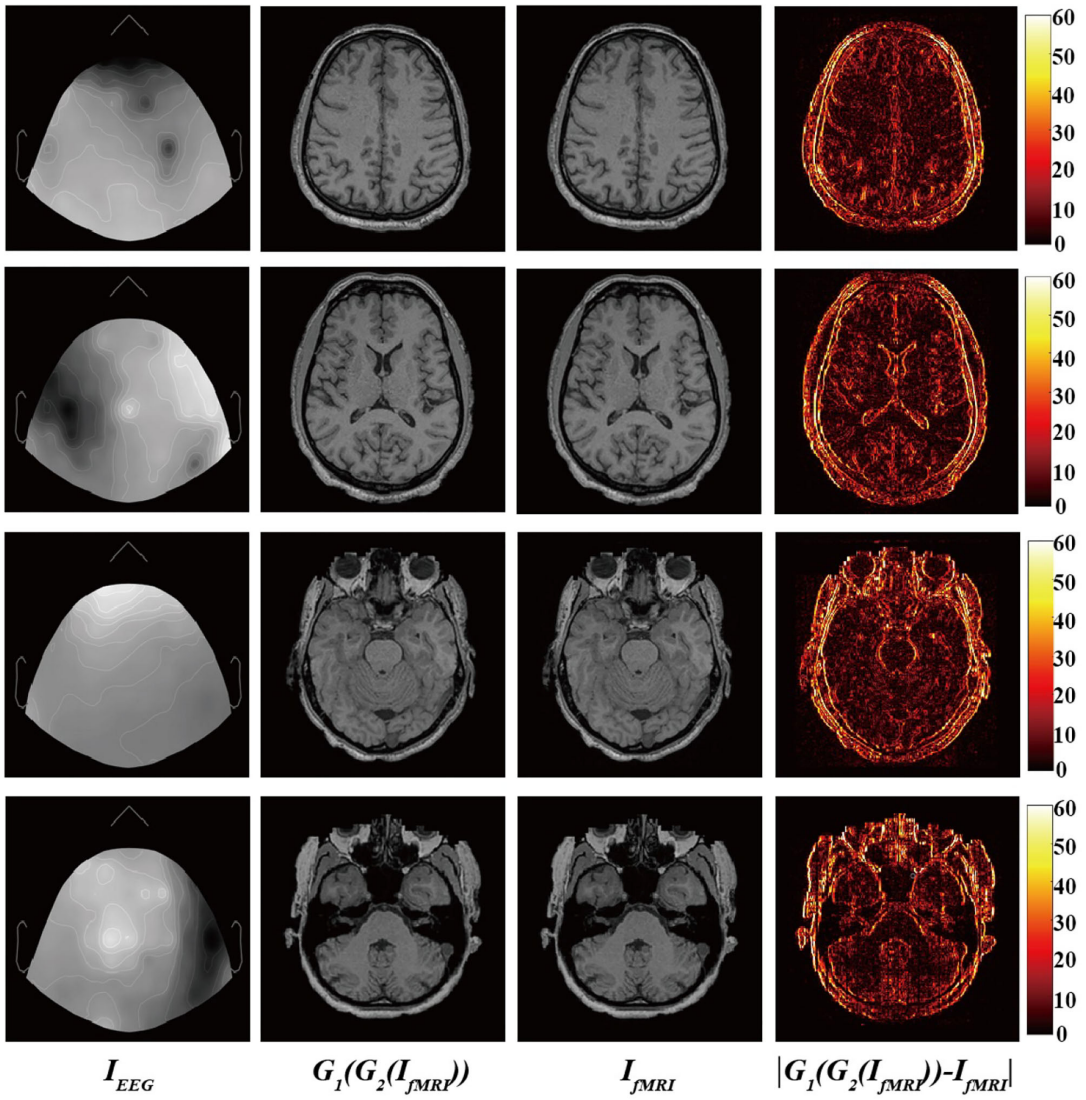
From **(left)** to **(right)**: Input EEG images, output transfer fMRI images, reference fMRI images, and absolute error map between the output translated fMRI and the reference fMRI images.

Finally, Figure 4 shows the input EEG image, the output fMRI image after modality transfer, the target fMRI image, and the absolute difference map between the output fMRI and the reference fMRI image.

It is worth mentioning that we narrowed the original difference range to [0,60] to make the absolute difference clearer and easier to analyze. Observed from local areas, there are still differences in some local areas. The biggest difference appears in the area of bone structure, while the smallest difference appears in the soft tissue area of brain tissue. This part may be caused by the misregistration between EEG and fMRI images and the large difference in image structure. From a holistic view, BMT-GAN has successfully learned different anatomical structures in fMRI images (such as bone, gyrus, and soft tissue of brain tissue) in EEG images, and learned to distinguish different anatomical structures with similar pixel intensity. In addition, BMT-GAN also implements the translation of EEG images to fMRI images and ensures the global consistency of the output fMRI images to the reference fMRI images.

### 3.2. State-of-the-Art Works

We compared BMT-GAN with the following methods:

(1) UNIT (Wang T.-C. et al., 2018): A method of using latent shared space for image transfer.

(2) CGAN (Mirza and Osindero, 2014; Bayramoglu et al., 2017): A method to generate the specified image. It adds additional information, such as labels, to the input of the original GAN generator and discriminator.

(3) pix2pix (Choi and Lee, 2018; Olut et al., 2018): A method uses a combination of L1 distance and antagonistic loss to train paired data.

(4) CycleGAN (Dar et al., 2019): This method learns two mappings, and introduces two Cycle-consistent loss and adversarial loss to regularize the mapping.

### 3.3. Evaluation Metrics

We use three commonly used image quality evaluation metrics: Mean-square error (MSE) (Chai and Draxler, 2014), Peak signal-to-noise ratio (PSNR) (De Boer et al., 2003), and Structural similarity (SSIM) (Wang et al., 2004). Their definitions are as follows:

(7) $$\begin{array}{l} MAE=\frac{1}{N}\overset{N}{\underset{i=1}{\sum }}|\left(I_{fMRI}\left(i\right)-G_{1}\left(I_{EEG}\left(i\right)\right)\right)| \end{array}$$

Where it is assumed that the number of 2D slices referred to in the fMRI image is N, i is the index of the image slices, and MAE is the measurement of the average distance between each pixel of the composite image and the real image. The PSNR formula is as follows:

(8) $$\begin{array}{l} MSE=\frac{1}{N}\overset{N}{\underset{i=1}{\sum }}{\left(I_{fMRI}\left(i\right)-G_{1}\left(I_{EEG}\left(i\right)\right)\right)}^{2} \end{array}$$

(9) $$\begin{array}{l} PSNR=10*log_{10}\left(\frac{MAX_{I_{fMRI}}^{2}}{MSE}\right) \end{array}$$

Where MAX is the maximum intensity value of the original image and the composite image. Since each pixel of the resulting composite image and the original image is represented by an 8-bit binary, *MAX* = 255. Both PSNR and MAE can be used to compare the performance of different models over a unified dataset, but they are limited only to image alignment. Therefore, we also need the structural similarity (SSIM) index, which can not only measure the pixel brightness difference of the image but also evaluate the contrast and structure of the image. Its formula is defined as:

(10) $$\begin{array}{l} \{\begin{array}{cc} L{\left(x,y\right)}_{i} & =\frac{2\mu_{i,x}\mu_{i,y}+C_{1}}{\mu_{i,x}^{2}+\mu_{i,y}^{2}+C_{1}}\\ C{\left(x,y\right)}_{i} & =\frac{2\sigma_{i,x}\sigma_{i,y}+C_{2}}{\sigma_{i,x}^{2}+\sigma_{i,y}^{2}+C_{2}}\\ S{\left(x,y\right)}_{i} & =\frac{\sigma_{i,xy}+C_{3}}{\sigma_{i,x}\sigma_{i,y}+C_{3}} \end{array} \end{array}$$

(11) $$\begin{array}{l} SSIM{\left(x,y\right)}_{i}=\left[L{\left(x,y\right)}_{i}^{\alpha }*C{\left(x,y\right)}_{i}^{\beta }*S{\left(x,y\right)}_{i}^{\gamma }\right] \end{array}$$

Set α, β, γ to 1, you can get:

(12) $$\begin{array}{l} SSIM{\left(x,y\right)}_{i}=\frac{\left(2\mu_{i,x}\mu_{i,y}+C_{1}\right)\left(2\sigma_{i,x}\sigma_{i,y}+C_{2}\right)}{\left(\mu_{i,x}^{2}+\mu_{i,y}^{2}+C_{1}\right)\left(\sigma_{i,x}^{2}+\sigma_{i,y}^{2}+C_{2}\right)} \end{array}$$

Where, μ_*i,x*_μ_*i,y*_ are the mean value of the synthetic image and the real image, respectively, and σ_*i,x*_σ_*i,y*_ are the synthetic image and the real image respectively The variance of the image, σ_*i,xy*_ is the covariance between the synthetic image and the real image, *C*_1_, *C*_2_, *C*_3_ are constants to avoid system errors caused by a denominator of 0.

The above three evaluation metrics measure the quality of the image from a statistical perspective by calculating the pixel error between the output image and the target image. These three evaluation metrics are relatively simple and easy to implement, but they do not take into account the local visual factors of human eyes, so there is no way to grasp the local quality of the image. Recent research has shown that Visual Information Fidelity (VIF) (Sheikh and Bovik, 2006) and Information Fidelity Criterion (IFC) (Sheikh et al., 2006) are widely used to evaluate image quality. They extend the link between the image and the human eyes in terms of fidelity of information. Therefore, we introduce these two valuation metrics, which make image similarity measurement more effective.

### 3.4. Result Analysis

The proposed BMT-GAN framework in this paper is experimentally verified. Compared with the traditional pix2pix framework, CGAN framework in the supervised field, and the famous CycleGAN, UNIT frameworks in the unsupervised field to prove the superiority of our proposed framework. Through the final five evaluation metrics and convergence performance to compare the similarity of pixel distribution histogram indicators to prove the feasibility and efficiency of BMT-GAN.

#### 3.4.1. Prediction Performance

**Figure 5:**
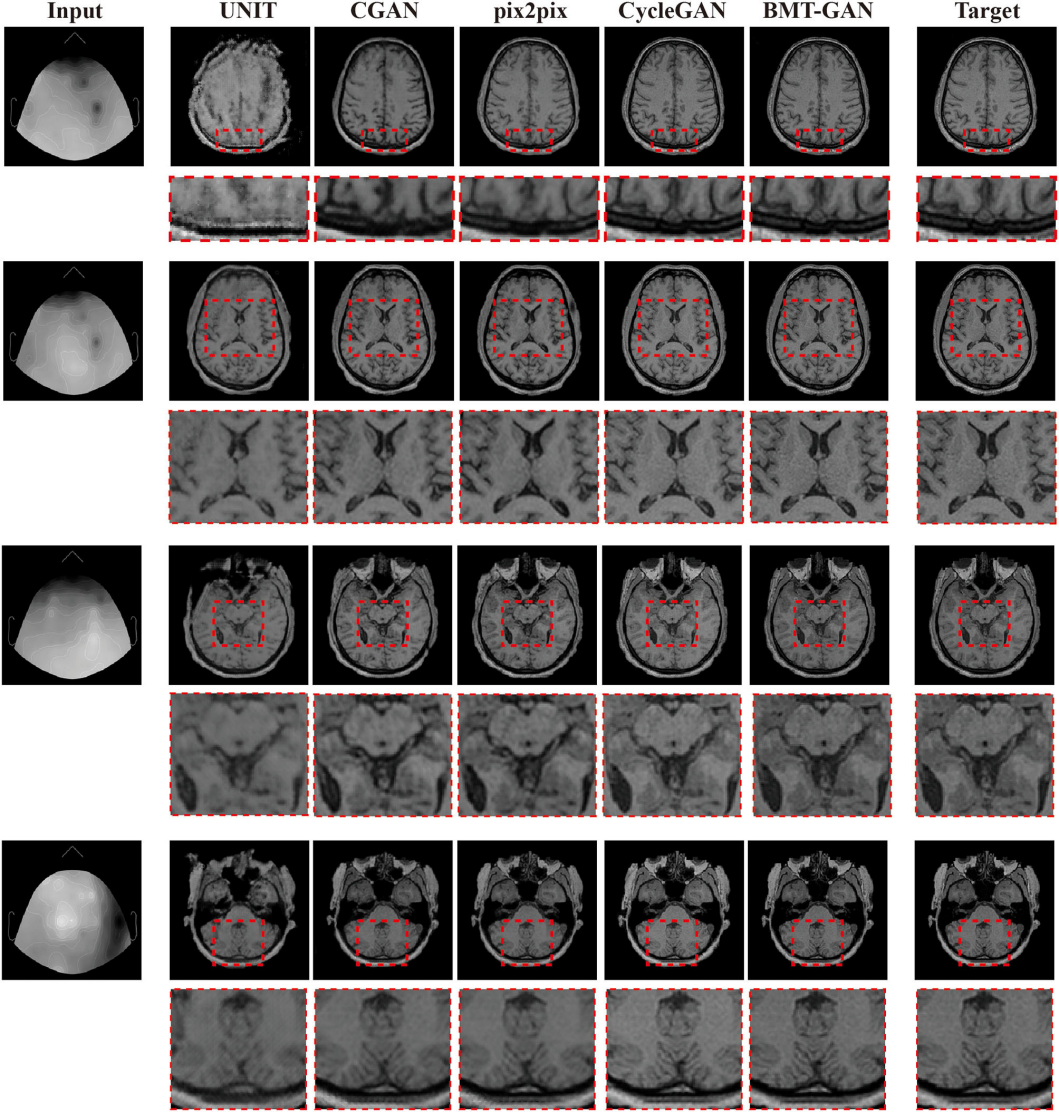
Qualitative comparisons between the BMT-GAN framework and other brain imaging modality transfer technologies. Describes the task of converting EEG to fMRI.

The experiment results are obtained by using target fMRI and output fMRI images as main examples. During training, the training data are completely consistent, and the same input is used for verification. The prediction results are shown in Figure 5. The UNIT framework completes the image transformation between the two domains, but distortion occurs in some areas of the image resulting in the blurred output of the translated image. Qualitatively, pix2pix and CGAN frameworks have also successfully achieved image conversion between two domains. It shows better performance than UNIT but lacks high resolution. The BMT-GAN framework and CycleGAN framework in this paper both have good results. However, the proposed BMT-GAN framework yields results that are closer to the ground truth, more closely connected between output, and input, twisted less and results not only on the style transformation is more apparent. The results show that the framework proposed in this paper has a good application value in the field of image translation.

In order to more accurately verify the performance of the proposed model. We introduce MSE, PSNR, SSIM, and other property price indicators to measure the output results through these numerical scoring standards. The specific results are shown in Table 1.

**Table 1:** Quantitative comparison of brain imaging modality transfer technologies.

**Method**	**MSE**	**PSNR**	**SSIM**	**VIF**	**IFC**
UNIT	1.0230e+03	18.0322	0.5705	0.0465	0.2733
CGAN	340.4902	22.8098	0.6783	0.1614	0.9207
pix2pix	241.0907	24.3090	0.8511	0.3392	2.3423
CycleGAN	143.9875	26.5476	0.8092	0.3323	2.1211
BMT-GAN	128.6233	27.0376	0.8627	0.3575	2.4794

Table 1 shows the MSE, PSNR, and other indicators values computed based on the predicted images with respect to the ground truth. Specifically, BMT-GAN again demonstrates the best performance among all the compared methods. BMT-GAN improves the state-of-the-art PSNR/SSIM performance from 26.5476/0.8092 given by CycleGAN to 27.0376/0.8627. However, as analyzed before, UNIT combined with traditional GAN networks and variational autoencoders to improve the output results, although very good results can be achieved, because two sets of GAN networks are trained at the same time, each step of training requires parameter updates for the two sets of GAN networks, resulting in higher training costs Large, the convergence rate is slower.

#### 3.4.2. Convergence Performance

**Figure 6:**
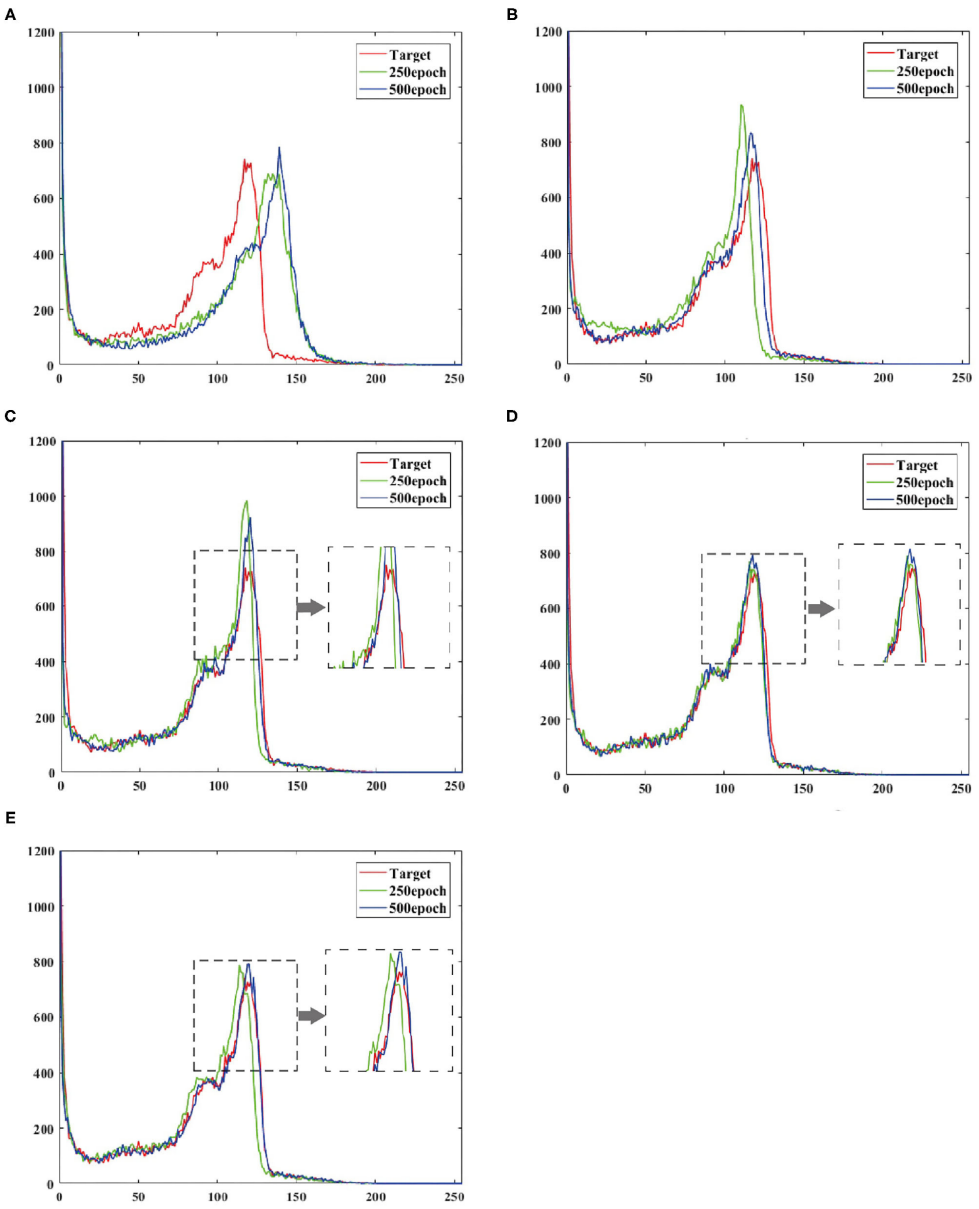
Shows the output reconstruction and the pixel distribution histogram of the target image at the 250th epoch, and the output reconstruction and the pixel distribution histogram of the target image at the 500th epoch. **(A)** UNIT, **(B)** CGAN, **(C)** pix2pix, **(D)** CycleGAN, **(E)** BMT-GAN.

To verify the effect of convergence speed on the model, we divided the training period of the model into 250 epochs and 500 epochs, and obtained the pixel distribution histogram of the reconstructed image and the target image at different epochs. Observe the distance between the pixel distribution histogram of different epochs and the target pixel distribution histogram. The size of the distance indicates how fast the model converges, as shown in Figure 6.

At the 250th epoch, we can see that the UNIT model has the slowest convergence speed, and the CycleGAN model has the fastest convergence speed. But because the histogram of the 500 epoch does not give clear results. Therefore, we use two commonly used evaluation indicators: Euclidean metric (EM) and cosine similarity (CS) to compare the similarity between the output reconstructed image of the five models and the pixel distribution histogram of the target image.

**Figure 7:**
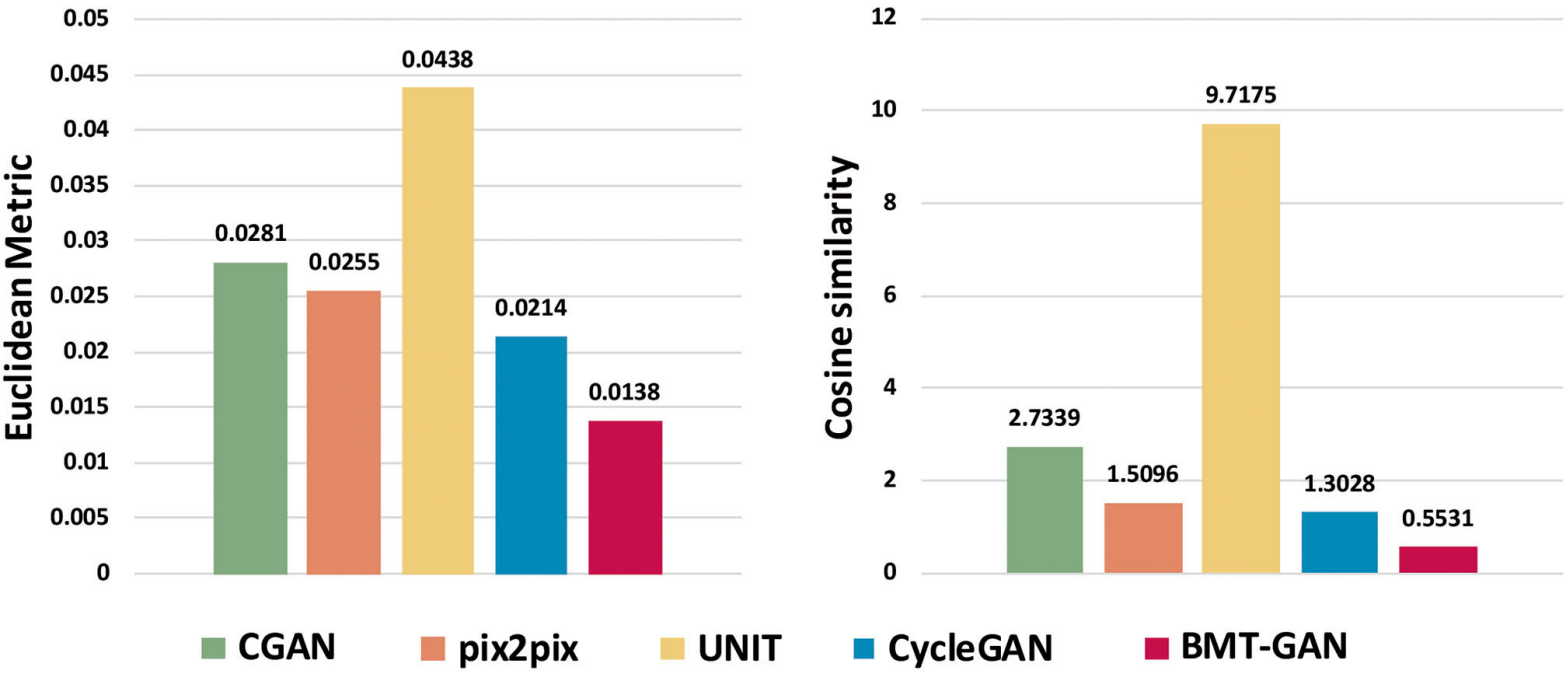
The output reconstruction of five different brain imaging modal transfer methods and the histogram of the Euclidean distance value and cosine similarity value of the pixel distribution histogram of the target image.

Table 2 and Figure 7 show that the model proposed in this paper is closest to the target value. Compared with other models, the BMT-GAN framework proposed in this paper can more effectively improve the performance of image translation tasks and enhance the relationship between output and input.

**Table 2:** Quantitative comparison of brain imaging modality transfer technologies.

**Method**	**UNIT**	**CGAN**	**pix2pix**	**CycleGAN**	**BMT-GAN**
EM	0.0438	0.0281	0.0255	0.0214	0.0138
CS	9.7175	2.7339	1.5096	1.3028	0.5531

## 4. Conclusion

This paper proposes a novel brain imaging modality transfer framework, namely BMT-GAN. The proposed BMT-GAN framework introduces the adversarial loss of discriminator network, dual-period consistent loss of Unpaired training data and the feature-based non-adversarial loss combination to realize the modality transformation from EEG image to fMRI image. Specifically, the traditional modality transfer methods of brain imaging are based on pixel loss, which usually leads to blurring results. In the field of medical images, small structures can significantly change the diagnostic information of images. Therefore, to capture the difference between the high-frequency components in the image, we introduce non-adversarial perception and style loss based on CycleGAN. This application can be said to be novel. It makes full use of the complementary information of different modality images and can synthesize fMRI images with better tissue contrast and anatomical details, which improves the diagnostic value of brain imaging technology in clinical medicine.

The qualitative and quantitative results show that the performance of BMT-GAN is superior to the existing methods of brain imaging modality transfer. Specifically, the output fMRI images were closer to the target, and the lowest MSE (128.6233), the highest PSNR (27.0367), and the highest SSIM (0.8627) were obtained. However, the deviation between EEG images and reference fMRI images may have a significant impact on quantitative evaluation. Although quantitative measurement is a standard to evaluate the performance of a method, in this case, the numerical difference in quantitative evaluation cannot correctly represent the quality difference. In future work, the accuracy of synthetic fMRI images based on medical expert perception studies will be evaluated.

## Data Availability Statement

The original contributions presented in the study are included in the article/supplementary material, further inquiries can be directed to the corresponding author/s.

## Author Contributions

All authors listed have made a substantial, direct and intellectual contribution to the work, and approved it for publication.

## Conflict of Interest

The authors declare that the research was conducted in the absence of any commercial or financial relationships that could be construed as a potential conflict of interest.
